# Bone marrow-derived macrophages from aged rats are more responsive to inflammatory stimuli

**DOI:** 10.1186/s12974-015-0287-7

**Published:** 2015-04-09

**Authors:** James P Barrett, Derek A Costello, Joan O’Sullivan, Thelma R Cowley, Marina A Lynch

**Affiliations:** Trinity College Institute for Neuroscience, Trinity College, Dublin 2, Ireland

**Keywords:** Age, Bone marrow-derived macrophages, Macrophage phenotypes, Lipopolysaccharide (LPS), Interferon-γ (IFNγ), Inflammatory cytokines

## Abstract

**Background:**

Lipopolysaccharide (LPS) and interferon-γ (IFNγ) increase expression of tumour necrosis factor-α (TNFα) that characterizes the M1 activation state of macrophages. Whereas it is accepted that the immune system undergoes changes with age, there is inconsistency in the literature with respect to the impact of age on the response of macrophages to inflammatory stimuli. Here, we investigate the effect of age on the responsiveness of bone marrow-derived macrophages (BMDMs) to LPS and IFNγ. The context for addressing this question is that macrophages, which infiltrate the brain of aged animals, will encounter the neuroinflammatory environment that has been described with age.

**Methods:**

Brain tissue, prepared from young and aged rats, was assessed for expression of inflammatory markers by PCR and for evidence of infiltration of macrophages by flow cytometry. BMDMs were prepared from the long bones of young and aged rats, maintained in culture for 8 days and incubated in the presence or absence of LPS (100 ng/ml) or IFNγ (50 ng/ml). Cells were harvested and assessed for mRNA expression of markers of M1 activation including TNFα and NOS2, or for expression of IFNγR1 and TLR4 by western immunoblotting. To assess whether BMDMs induced glial activation, mixed glial cultures were incubated in the presence of conditioned media obtained from unstimulated BMDMs of young and aged rats and evaluated for expression of inflammatory markers.

**Results:**

Markers associated with M1 activation were expressed to a greater extent in BMDMs from aged rats in response to LPS and IFNγ, compared with cells from young rats. The increased responsiveness was associated with increases in IFNγ receptor (IFNγR) and Toll-like receptor 4 (TLR4). The data show that conditioned media from BMDMs of aged rats increased the expression of pro-inflammatory mediators in glial cells. Significantly, there was an age-related increase in macrophage infiltration into the brain, and this was combined with increased expression of IFNγ and the Toll-like receptor 4 agonist, high-mobility group protein B1 (HMGB1).

**Conclusion:**

Exposure of infiltrating macrophages to the inflammatory microenvironment that develops in the brain with age is likely to contribute to a damaging cascade that negatively impacts neuronal function.

## Introduction

Macrophages are key cells in driving the innate immune response and form a heterogenous population that possesses tissue-specific roles. These cells display remarkable plasticity with functions that include the initiation and resolution of changes that occur in response to inflammatory stimuli, phagocytosis, maintenance of tissue homeostasis and tissue remodelling and repair [1]. Bone marrow-derived macrophages (BMDMs) are derived from stem cells that can differentiate into mature macrophages in the presence of growth factors *in vitro*. Similar to tissue macrophages, they have the ability to respond to a wide array of stimuli. The classical activation state, also called M1, describes a shift in the cell from a quiescent to a pro-inflammatory state and can be triggered by interferon (IFN)γ or activation of Toll-like receptors (TLRs), particularly TLR4. These cells are typified by upregulation of expression of nitric oxide synthase (NOS2) and inflammatory cytokines like tumour necrosis factor (TNF)α [2]. In response to anti-inflammatory cytokines such as interleukin (IL)-4, macrophages adopt an alternative activation state, also called M2, as originally described in the early 1990s [3]; this phenotype is important in regulating inflammation, mediating tissue repair and restoring homeostasis. M2 macrophages are characterized by increased expression of arginase-1 (Arg-1), mannose receptor (MRC1), chitinase 3-like 3 (Chi3l3) and found in inflammatory zone 1 (Fizz1) [4,5].

The immune system undergoes changes with age and, in the context of macrophages, it has been suggested that oxidative damage to DNA contributes to their age-related dysfunction [6]. It has been reported that the IFNγ-induced respiratory burst, which accompanies phagocytosis, is reduced in peritoneal macrophages from aged animals [7]. However, recent evidence revealed that there was an age-related increase in lipopolysaccharide (LPS)-induced NO production and increases in expression of proteins that are associated with phagocytosis and antigen presentation coupled with a decrease in markers of alternative activation [8]. It has been suggested that macrophages from aged animals respond less well, in terms of inflammatory cytokine production, to stimuli like LPS [9], but there is evidence that age-related changes in macrophage function are tissue-specific. For example, an increase in inflammatory cytokine production in LPS-treated alveolar macrophages from aged animals has been demonstrated, but a decrease was observed in peritoneal macrophages [10]. The effect of LPS on expression of NOS2 and IL-6 mRNA was decreased in splenocytes prepared from aged mice [11,12], while IL-10 was increased perhaps as a consequence of increased signaling through Akt [13]. Interestingly, splenocytes from aged mice were refractory to IL-4 stimulation, possibly because of a decrease in IL-4 receptor expression [11]. There is also evidence of age-related changes in BMDMs; for example, granulocyte macrophage-colony stimulating factor (GM-CSF)-dependent proliferation was decreased with age and this was attributed to telomere loss coupled with enhanced oxidative stress, although dysregulation of signaling through signal transduction and transcription (STAT)5a was also observed [6]. Additionally, the IL-1β released in response to LPS + ATP was attenuated in cells from aged mice. This resulted from altered processing of pro-IL-1β to the mature form, although inflammasome assembly in BMDMs was not affected [14]. In contrast, IFNγ exerted similar effects on BMDMs from aged and young mice [15].

The responsiveness of BMDMs to inflammatory stimuli is significant given that macrophages infiltrate the brain in conditions which are characterized by neuroinflammation. For example, an increase in BMDMs in the brain has been documented in animal models of stroke [16] and encephalitis [17]. In addition, we have recently reported an age-related increase in macrophage number in the brain of middle-aged mice [18], mice which overexpress amyloid precursor protein (APP) and presenilin 1 (PS1; APP/PS1 mice) [18,19] and CD200-deficient mice [20], which also exhibit increases in expression of inflammatory markers. The role these macrophages play within the brain is still undetermined, but it is possible that these cells contribute to the existing neuroinflammatory environment.

The objective of this study was to evaluate the effect of age on the response of BMDMs to LPS and IFNγ and to establish whether conditioned medium from macrophages impacts on glial activation. The data indicate that the cells from aged rats were hyper-responsive to LPS and IFNγ, and this is significant since macrophages infiltrate the brain with age and encounter an inflammatory microenvironment that could activate the cells. Conditioned media from BMDMs of aged rats increased the expression of pro-inflammatory mediators in glial cells; therefore, we propose that, in the CNS of aged animals, infiltration of macrophages propagates the existing inflammation.

## Materials and methods

### Animals

Male Wistar rats were used in this study. Animals were obtained from B & K Universal (North Humberside, UK) and maintained under veterinary supervision in a controlled environment (temperature: 20°C to 22°C; 12:12-h light/dark cycle) in the Bioresources Unit in Trinity College, Dublin, for the duration of the study. Rats had free access to food and water. All procedures were carried out under licence from the Health Products Regulatory Authority of Ireland in accordance with EU regulations and with ethical approval obtained from the local ethics committee.

Animals were anaesthetized with urethane (1.5 mg/kg) and transcardially perfused with ice-cold phosphate-buffered saline (PBS). Brains were rapidly removed, placed on ice and hemisected. The hippocampus and cortex were dissected free from one half of the brain and stored for later analysis of RNA and protein, and a single cell suspension was prepared from the other half of the brain for analysis of infiltrating macrophages by flow cytometry (see below). The legs were sprayed with 70% ethanol, and the tibiae and femurs removed for the preparation of BMDMs.

### Preparation, culture and treatments of BMDMs

BMDMs were isolated from the marrow of the femurs and tibias of 3-month-old (200 to 360 g) and 20- to 27-month-old rats. The legs of the animals were sprayed with 70% EtOH, and the skin and muscle tissue were removed from the bones. The bones were sprayed with 70% EtOH, transferred to a sterile-flow hood and cut at both ends. The marrow was flushed out into a sterile falcon tube in Dulbecco’s modified Eagle’s medium (DMEM; 500 ml; Invitrogen, Renfrew, UK) supplemented with heat-inactivated foetal bovine serum (FBS; 50 ml; 10%; Gibco, Paisley, UK) and penicillin-streptomycin (5 ml; 1%; Gibco, Paisley, UK). The cell suspension was triturated using a sterile Pasteur pipette, filtered through a nylon mesh filter (40 μm; BD Biosciences, Oxford, UK) into a sterile tube and centrifuged (400 × *g*, 5 min). The supernatant was removed, and the pellet was resuspended in red blood cell lysis buffer (Sigma-Aldrich, Gillingham, Dorset, UK). The suspension was centrifuged (400 × *g*, 5 min), the supernatant was discarded and the cells were washed using DMEM and centrifuged once more (400 × *g*, 5 min). The pellet was resuspended in 20 ml of DMEM supplemented with L929-conditioned media (20%).

Cells were seeded in sterile cell culture flasks (T175 cm^2^ flasks). On day 2, non-adherent cells were removed from the flask, the media was replaced and the remaining adherent cells were maintained in culture for a further 6 days, with media being replaced on day 4. On day 6, cells were transferred to 6-well plates (0.5 × 10^6^ cells per well) or 24-well plates (0.4 × 10^6^ cells per well) and cultured for another 2 days, after which time they were incubated in the presence or absence of LPS (100 ng/ml) or IFNγ (50 ng/ml) for 3, 6 or 24 h. Supernatant samples were collected for analysis of cytokines by ELISA or for use as conditioned media, and cells were harvested for analysis of markers of macrophage activation states by real-time PCR.

### Macrophage infiltration assessed by flow cytometry

Mononuclear cells were isolated from brain tissue of rats aged 3 to 4 months and 16 months of age. Briefly, Stock Isotonic Percoll (SIP) was obtained by mixing nine volumes of Percoll (Sigma-Aldrich, Gillingham, Dorset, UK) with one volume of × 10 PBS. Further Percoll gradients were prepared with × 1 PBS as appropriate. Following sacrifice, brain tissue was dissected and placed in × 1 HBSS (Invitrogen, Renfrew, UK), cross-chopped, homogenized and triturated using fire-polished Pasteur pipettes with three decreasing diameters. Cell suspensions were filtered through a cell strainer (70 μm) and cells pelleted by centrifugation. The resultant pellets were resuspended in 75% Percoll (10 ml) overlaid with 25% Percoll (10 ml) and × 1 PBS (6 ml) and centrifuged at 800 × *g* for 30 min at 4°C. Following centrifugation, an enriched mononuclear cell population was collected from the 25% to 75% interface. Infiltrating macrophages were identified by staining for PE-Cy7 anti-rat CD45 (1:100, BD Bioscience, Oxford, UK) and Alexa Fluor 647 anti-rat CD11b (1:100; Serotec, Kidlington, UK). A forward- versus side-scatter gating strategy was employed to exclude debris and dead cells which have characteristically low forward- and high side-scatter signals. Infiltrating macrophages were identified as CD11b^+^CD45^high^ cells, a strategy commonly employed to identify these cells [21-24]. The data is expressed as the percentage of CD11b^+^CD45^high^ cells present. Immunofluorescence was read immediately on a DAKO CyAn-ADP 7 colour flow cytometer with Summit software v4.3 for acquisition. BD™ CompBeads (BD Biosciences;, Oxford, UK) were used to optimize fluorescence settings and further flow cytometric analysis was carried out in FlowJo v7.6.5. Unstained cells and fluorescence minus one (FMO) tubes were used to gate the percentage of positive cells in any channel.

### Preparation of primary glial cultures

Mixed glial cultures were prepared from the cortices of 1-day-old Wistar rats (Trinity College, Dublin, Ireland). Cortical tissue was cross-chopped, incubated for 25 min at 37°C in DMEM (Invitrogen, Renfrew, UK) supplemented with 10% foetal bovine serum (Invitrogen, Renfrew, UK) and 50 U/m penicillin/streptomycin (Invitrogen, Renfrew, UK) and plated (1 × 10^4^/cm^2^) as previously described [25]. After 12 days in culture, cells were incubated with conditioned media from BMDMs cultured from young and aged animals (as described above) for 24 h, and the cells were harvested for analysis of mRNA.

### Quantitative real-time PCR

RNA was isolated from cultured BMDMs, glial cultures and hippocampal tissue using the Nucleospin® RNAII KIT (Macherey-Nagel, Duren, Germany), and cDNA was prepared using High-Capacity cDNA RT kit according to the manufacturer’s instructions (Applied Biosystems, Warrington, UK). Real-time PCR for the detection of CD40, CD11b, MHCII, ICAM-1, IP-10, MCP-1, NOS2 and TNFα mRNA was performed with predesigned Taqman gene expression assays (Applied Biosystems, Warrington, UK). The assay IDs were as follows: CD40 (Rn1423590_m1), CD11b (itgam) (Rn00709342_m1), MHCII (Rn01768597_m1), NOS2 (Rn00561646_m1), TNFα (Rn00562055_m1), IFNγ (Rn00594078_m1), MCP-1 (Rn00580555_m1), ICAM-1(Rn64227_m1) and IP-10 (Rn00594648_m1). All real-time PCRs were conducted using an Applied Biosystems 7500 Fast Real-Time PCR machine (Applied Biosystems, Warrington, UK). Samples were assayed in one run (40 cycles), which consisted of three stages, 95°C for 10 min, 95°C for 15 s for each cycle (denaturation) and finally the transcription step at 60°C for 1 min. β-actin was used as endogenous control to normalize gene expression data, and β-actin expression was assessed using a gene expression assay containing forward and reverse primers (primer limited) and a VIC-labelled MGB Taqman® probe (Assay ID: 4352340E; Applied Biosystems, Warrington, UK). Gene expression was calculated relative to the endogenous control samples and to the control sample giving an RQ value (2^−DDCt^, where CT is the threshold cycle).

### Analysis of TNFα by ELISA

The concentration of TNFα in supernatant samples from cultured BMDMs was assessed by ELISA as previously described [26]. Briefly, 96-well plates (Nunc-Immuno plate with Maxisorp surface, Denmark) were coated with capture antibody (goat anti-mouse TNFα antibody (0.8 μg/ml in PBS; BD Biosciences, Oxford, UK)) and incubated (overnight, 4°C). Duplicate samples or standards (50 μl) were added and plates were incubated (24 h, 4°C) and washed before addition of detection antibody (200 ng/ml in PBS containing 10% FBS; 1 h, room temperature). Plates were washed again, incubated with streptavidin-horseradish peroxidase conjugate (50 μl; 1:200; 20 min, room temperature) and washed before addition of substrate solution (50 μl; 1:1 H_2_O_2_:tetramethylbenzidine; R&D Systems, Minneapolis, MN, US). After colour development, the reaction was stopped by adding 1 M H_2_SO_4_ (25 μl), and plates were read at 450 nm (Labsystem Multiskan RC, Thermoscientific, Waltham, MA, US).

### Analysis of proteins by western immunoblotting

Hippocampal lysate was assessed for expression of high-mobility group protein B1 (HMGB1); BMDM lysate was evaluated for expression of TLR4 and IFNγ receptor (IFNγR1). Briefly, samples were equalized for protein concentration, boiled in gel-loading buffer and separated by gel electrophoresis on 10% sodium dodecyl sulphate-polyacrylamide gels. Proteins were transferred to nitrocellulose membranes and incubated with anti-HMGB-1 antibody (anti-rabbit 1:2,000; Abcam, Cambridge, UK), TLR4 (anti-rabbit 1:1,000; Abcam, Cambridge, UK), IFNγR (anti-rabbit 1:1,000; Abcam, Cambridge, UK) or anti-β-actin antibody (mouse monoclonal; 1:5,000; Sigma-Aldrich, Gillingham, Dorset, UK). Membranes were incubated with horseradish peroxide-conjugated secondary antibodies (1:5,000; Jackson Immunoresearch, West Grove, PA, USA), and bands were visualised using WesternBright ECL Substrate (Advansta, Menlo Park, CA, USA). Images were captured using a Fujifilm LAS-4000 (Brennan and Co, Dublin, Ireland). Densitometry was performed using ImageJ software (http://rsb.info.nih.gov/ij/).

### Statistical analysis

Data are reported as the mean ± SEM, and the number of experiments is indicated in each case. Statistical analysis was carried out using a two-way analysis of variance (ANOVA), with *post hoc* Bonferroni tests or, where appropriate, one-way ANOVA followed by *post hoc* Newman-Keuls analysis to identify specific differences between groups. When comparisons were being made between two conditions, an unpaired Student’s *t*-test was performed. Significance level was set as *P* < 0.05.

## Results

**Figure 1:**
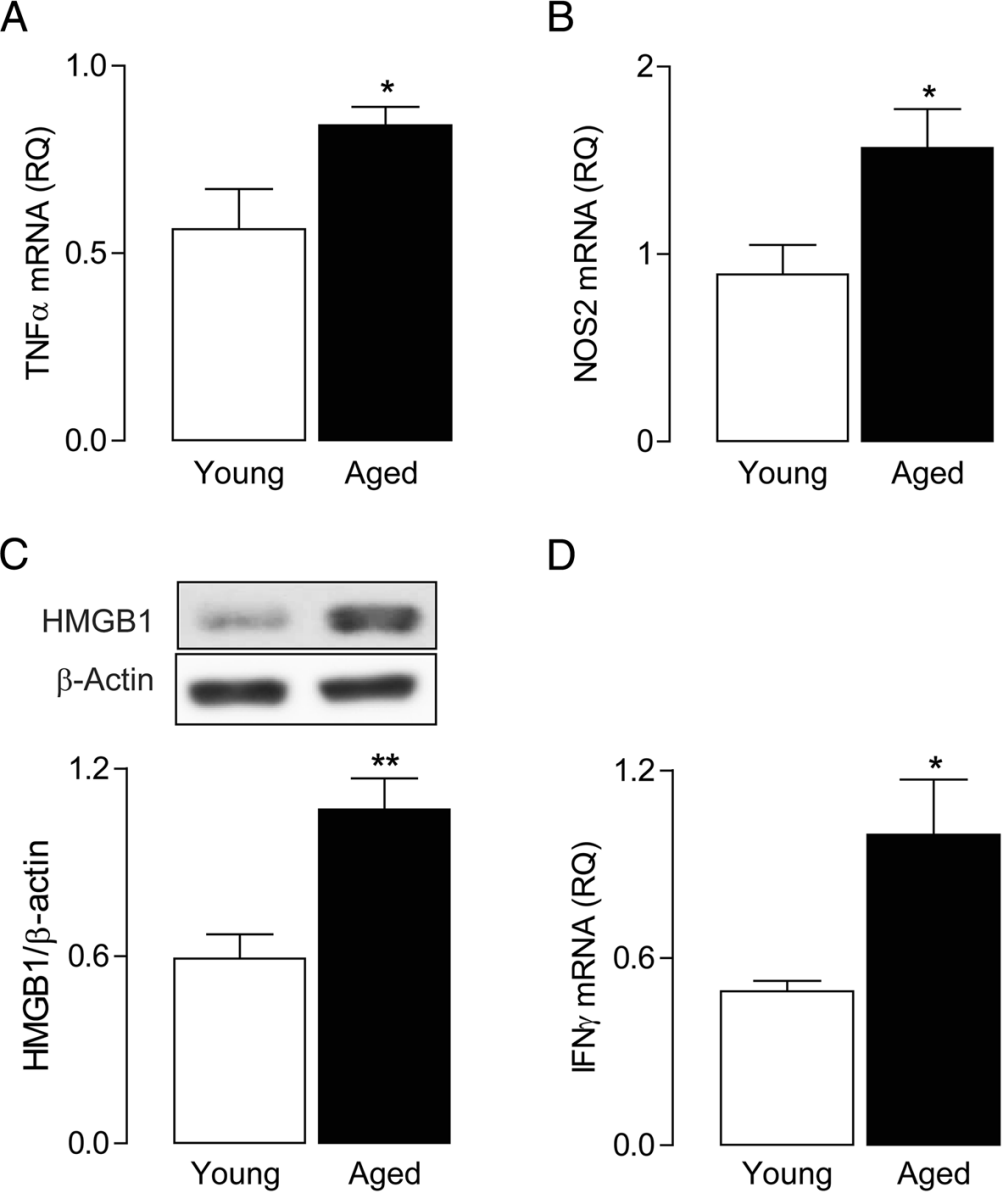
**Enhanced pro-inflammatory profile in the aged brain.** Hippocampal expression of TNFα **(A)**, NOS2 **(B)** and IFNγ **(D)** mRNA was significantly greater in aged rats (^*^ *P* < 0.05; Student’s *t-*test; *n* = 5 to 15). Age was also associated with an increase in HMGB1 **(C)** protein expression in hippocampal tissue (^**^ *P* < 0.01; Student’s *t-*test; *n* = 5 to 7). For mRNA data, values are presented as means (±SEM) and expressed as a ratio to β-actin mRNA. HMGB1, high-mobility group protein B1; IFNγ, interferon-γ; NOS2, nitric oxide synthase; RQ, relative quantities; TNFα, tumour necrosis factor-α.

Evidence of enhanced inflammatory activity within the aged brain has been previously reported, and consistent with previous studies [27-29], age-related increases in hippocampal mRNA expression of the two archetypal M1 markers, TNFα and NOS2, were observed (^*^P < 0.05; Student’s *t*-test for independent means; Figure 1A, B). These changes were accompanied by increased hippocampal concentration of the TLR2 and 4 agonist, HMGB1 and expression of IFNγ mRNA (^**^P < 0.01; ^*^P < 0.05; Student’s *t*-test for independent means; Figure 1C, D).

**Figure 2:**
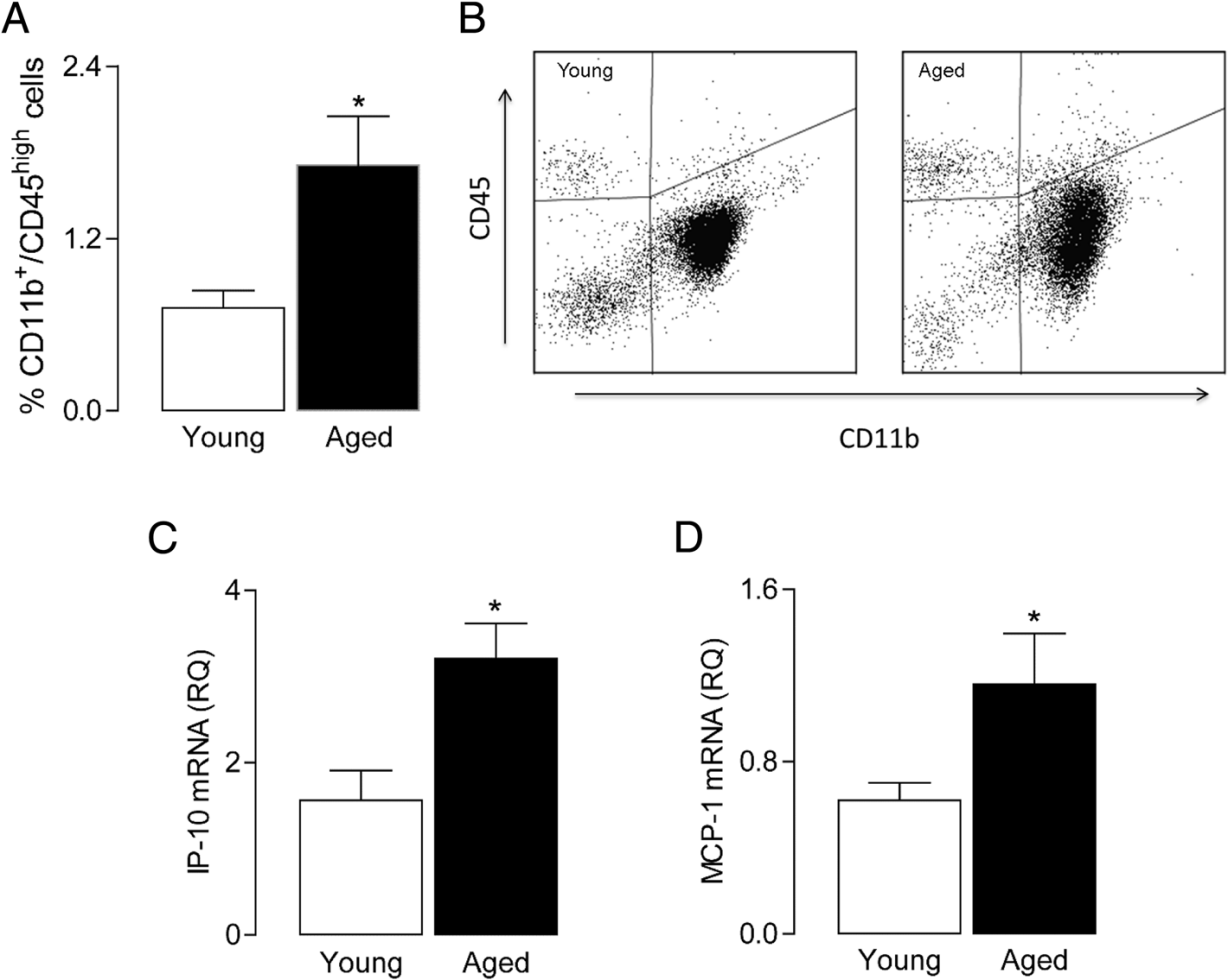
**Age is associated with increased macrophage infiltration and chemokine expression.** There was a significant increase in the percentage of macrophages **(A)** in the brains of older rats (^*^ *P* < 0.05; Student’s *t-*test; *n* = 4); values are presented as means (±SEM). **(B)** A representative plot of the flow cytometry data. There was an age-related increase in IP-10 **(C)** and MCP-1 **(D)** mRNA expression in hippocampal tissue (^*^ *P* < 0.05; Student’s *t-*test; *n* = 6 to 15). For mRNA data, values are presented as means (±SEM) and expressed as a ratio to β-actin mRNA. IP-10, interferon gamma-induced protein 10; MCP-1, monocytes chemotactic protein 1; RQ, relative quantities.

Blood brain barrier permeability is increased with age [30,31], and in APP/PS1 mice at least, this is associated with infiltration of peripheral cells including macrophages [31]. We considered that peripheral cells may also infiltrate the rat brain with age, and the data obtained from flow cytometric analysis indicate an increase in CD11b^+^CD45^high^ macrophages in tissue prepared from aged, compared with young, rats (^*^P < 0.05; Student’s *t*-test for independent means; Figure 2A). Whereas increased blood brain barrier permeability is likely to facilitate infiltration of peripheral cells into the brain, chemokine expression in the brain is also a contributory factor, and here, we show an age-related increase in hippocampal expression of both MCP-1 and IP-10 (^*^P < 0.05; Student’s *t*-test for independent means; Figure 2B, C), which are chemotactic for a number of cells including monocytes/macrophages [32].

**Figure 3:**
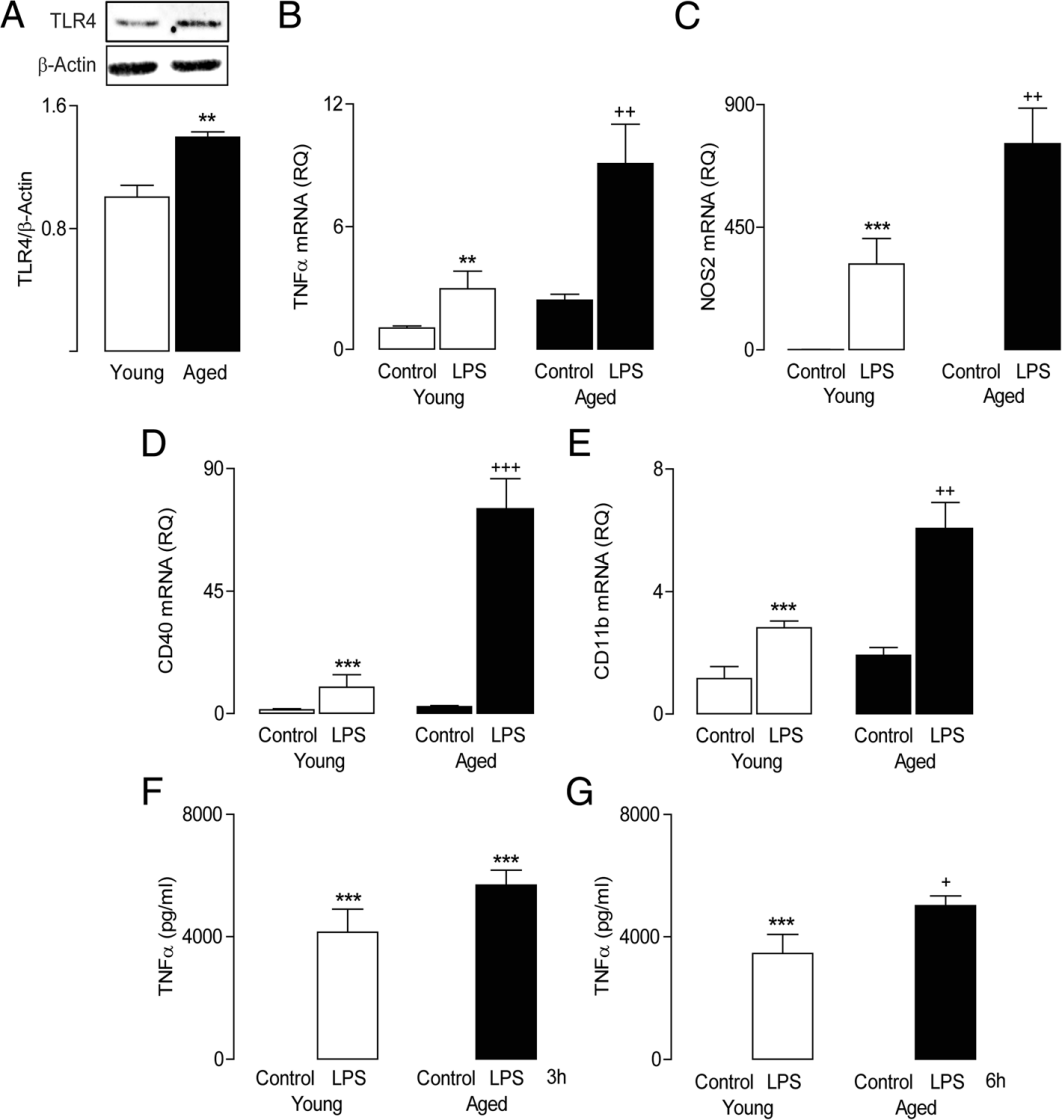
**Age is associated with increased TLR4 expression and enhanced sensitivity to LPS.** TLR4 **(A)** protein expression (assessed by western immunoblot) in BMDMs was found to be increased in an age-related manner (^**^ *P* < 0.01; Student’s *t-*test; *n* = 3). LPS (100 ng/ml; 24 h) stimulation significantly increased TNFα **(B)**, NOS2 **(C)**, CD40 **(D)** and CD11b **(E)** mRNA in BMDMs from young and aged rats (^***^ *P* < 0.001; ^**^ *P* < 0.01; ANOVA); the LPS-induced increase in mRNA expression of these markers was significantly greater in BMDMs cultured from aged, compared with young, rats (^++^ *P* < 0.01; ^+++^ *P* < 0.001; ANOVA). LPS stimulation (100 ng/ml; 3 and 6 h) significantly increased supernatant concentration of TNFα (measured by ELISA) in BMDMs from young and aged rats **(F, G)**. While significant TNFα release was identified in cells from young and aged animals (F, G; ^***^ *P* < 0.001, ANOVA), this LPS effect was significantly greater in BMDMs from aged rats at 6 h (G; ^+^ *P* < 0.05, ANOVA). Values are presented as means (±SEM; *n* = 3) expressed as a ratio to β-actin mRNA or as pg/ml. LPS, lipopolysaccharide; NOS2, nitric oxide synthase 2; CD40, cluster of differentiation 40; CD11b, cluster of differentiation 11b; TNFα, tumour necrosis factor-α; RQ, relative quantities.

Given the age-related increase in macrophage infiltration, and the fact that these cells encounter M1 polarizing stimuli, including HMGB1, we set out to compare the responses of BMDMs prepared from young and aged rats to another TLR4 agonist, LPS. An age-related increase in TLR4 expression was observed in BMDMs prepared from aged, compared with young, rats (^*^P < 0.05; Student’s *t*-test; Figure 3A). BMDMs exposed to LPS for a period of 24 h exhibited an increase in the mRNA expression of a number of markers associated with the M1 phenotype. LPS significantly increased mRNA expression of TNFα (^**^P < 0.01; ANOVA; Figure 3B) and NOS2 (^***^P < 0.01; ANOVA; Figure 3C), and the LPS-induced effect was significantly greater in BMDMs from aged rats (^++^p < 0.01; ANOVA; LPS-induced change in cells from aged *vs* young rats; Figures 3B, C). LPS significantly increased mRNA expression of CD40 (Figure 3D) and CD11b (Figure 3E) in BMDMs from young rats (^***^P < 0.001; ANOVA), and this effect was enhanced in cells from aged animals (^++^*P* < 0.01; ^+++^*P* < 0.01; LPS-induced change in cells from aged *vs* young rats; ANOVA). Two-way ANOVA revealed that incubation of cells with LPS for 3 or 6 h significantly increased supernatant concentration of TNFα (measured by ELISA) in BMDMs from young and aged animals (***P < 0.001, ANOVA; Figure 3F, G) which, following 6 h LPS exposure, was further enhanced in cells from aged rats (^+^*P* < 0.05, ANOVA; Figure 3G).

**Figure 4:**
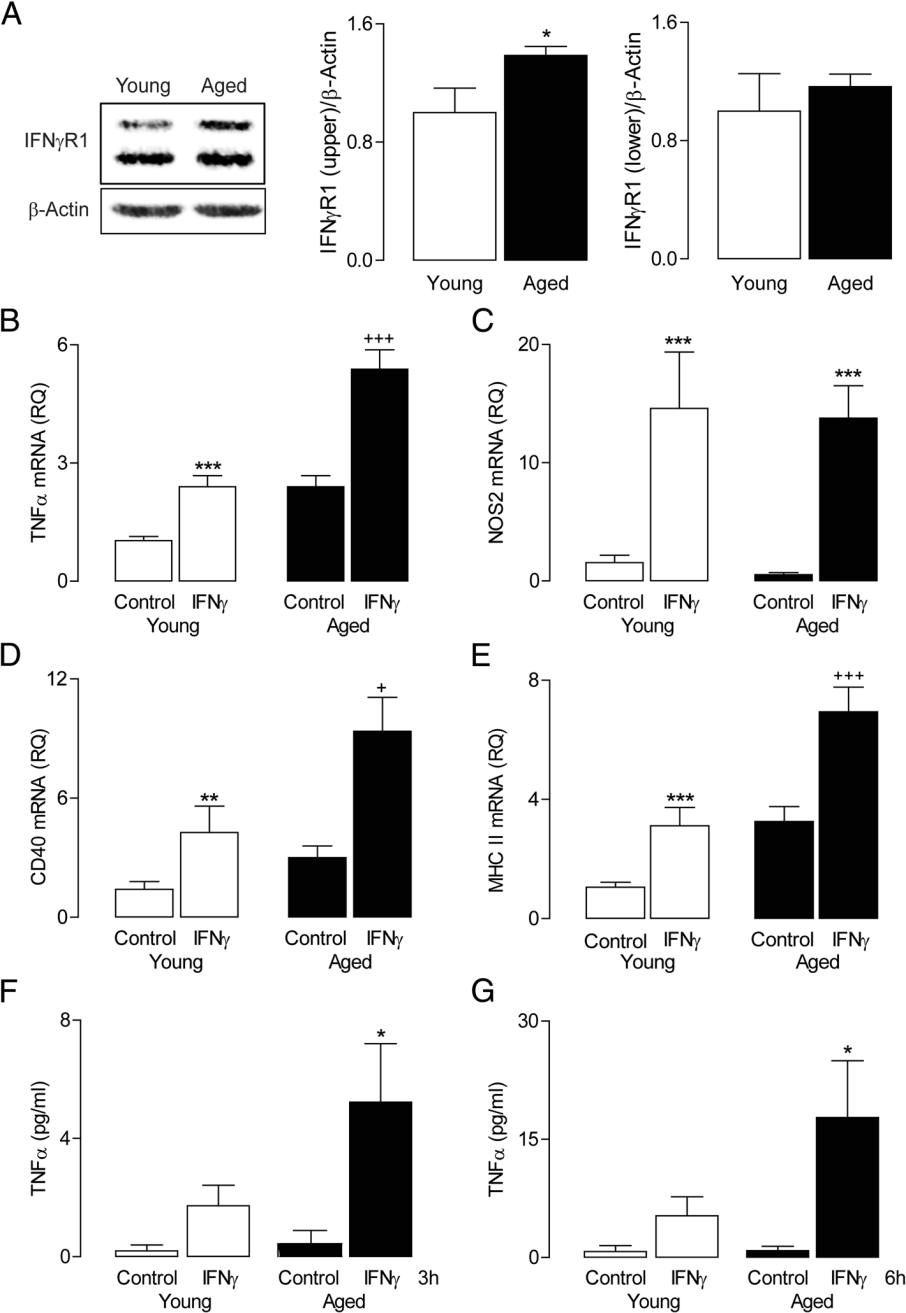
**IFNγ induces NOS2 and TNFα mRNA expression in macrophages from young and aged rats.** IFNγR1 **(A)** protein expression (assessed by western immunoblot) was found to be greater in BMDMs prepared from aged compared with young rats (^*^ *P* < 0.05, Student’s *t*-test; *n* = 3). IFNγ stimulation (50 ng/ml; 24 h) significantly increased TNFα **(B)**, NOS2 **(C)**, CD40 **(D)** and MHCII **(E)** mRNA in BMDMs from young and aged rats (^***^ *P* < 0.001; ANOVA). The IFNγ-induced increase in TNFα, CD40 and MHCII mRNA was significantly greater in BMDMs cultured from aged rats compared to cells derived from young rats (^++^ *P* < 0.01; ^+++^ *P* < 0.001; ANOVA). **(F, G)** BMDMs were treated with IFNγ (50 ng/ml) for 3 and 6 h; following this, TNFα supernatant concentration was measured by ELISA. The IFNγ-induced TNFα release from BMDMs only reached significance in cells from aged rats following 3- and 6-h incubation (*P < 0.05, ANOVA; n = 3). Values are presented as means (±SEM; n = 3) expressed as a ratio to β-actin mRNA or as pg/ml. IFNγ, interferon-γ; IFNγR1, IFNγ receptor; NOS2, nitric oxide synthase; CD40, cluster of differentiation 40; MHCII, major histocampatability complex II; TNFα, tumour necrosis factor-α; RQ, relative quantities.

Given the age-related increase in mRNA expression of IFNγ in the hippocampus, and since macrophages gain entry to the brain, the response of BMDMs to IFNγ stimulation was assessed. Western immunoblot analysis of unstimulated BMDMs with α-IFNγR1 antibody revealed two distinct immunoreactive bands (Figure 4A). While the lower band displayed equal abundance in cells from young and aged animals, the upper band showed a significant age-related increase (*P < 0.05, one-tailed Student’s *t*-test; Figure 4A). IFNγ stimulation (24 h) increased mRNA expression of TNFα and NOS2 in BMDMs prepared from young rats (^***^P < 0.001; ANOVA; Figure 4B, C), and the effect on TNFα mRNA, but not NOS2 mRNA, was further enhanced in BMDMs from aged rats (^+++^*P* < 0.001; IFNγ-induced change in cells from aged *vs* young rats). Similarly, the significant IFNγ-induced increases in mRNA expression of CD40 and MHCII (^**^P < 0.01; ^***^P < 0.001; ANOVA; Figure 4D, E) was accentuated in BMDMs from aged rats (^+^*P* < 0.05; ^+++^*P* < 0.001; IFNγ-induced change in cells from aged *vs* young rats). Two-way ANOVA revealed an IFNγ-induced increase in supernatant TNFα concentration (measured by ELISA) after incubation for 3 or 6 h, and this was significant in the cells from aged, but not from young, rats (*P < 0.05; Figure 4F, G).

While the data presented here focuses on the responses of BMDMs to pro-inflammatory stimuli, we also assessed the effect of IL-4 on mRNA expression of MRC1 and Arg-1. IL-4 increased mRNA expression of MRC1 (1.84 ± 0.25 (IL-4) *vs* 1.03 ± 0.13 (control); *P* < 0.01) and Arg-1 (16.38 ± 2.73 (IL-4) *vs* 1.51 ± 0.58 (control); *P* < 0.001) in BMDMs prepared from young animals. The effect of IL-4 on MRC1 was significantly reduced in BMDMs from aged (1.18 ± 0.14), compared with young (1.84 ± 0.25), rats, but the effect of IL-4 on Arg-1 was similar in BMDMs from aged (20.74 ± 3.3) and young (16.38 ± 2.74) rats.

**Figure 5:**
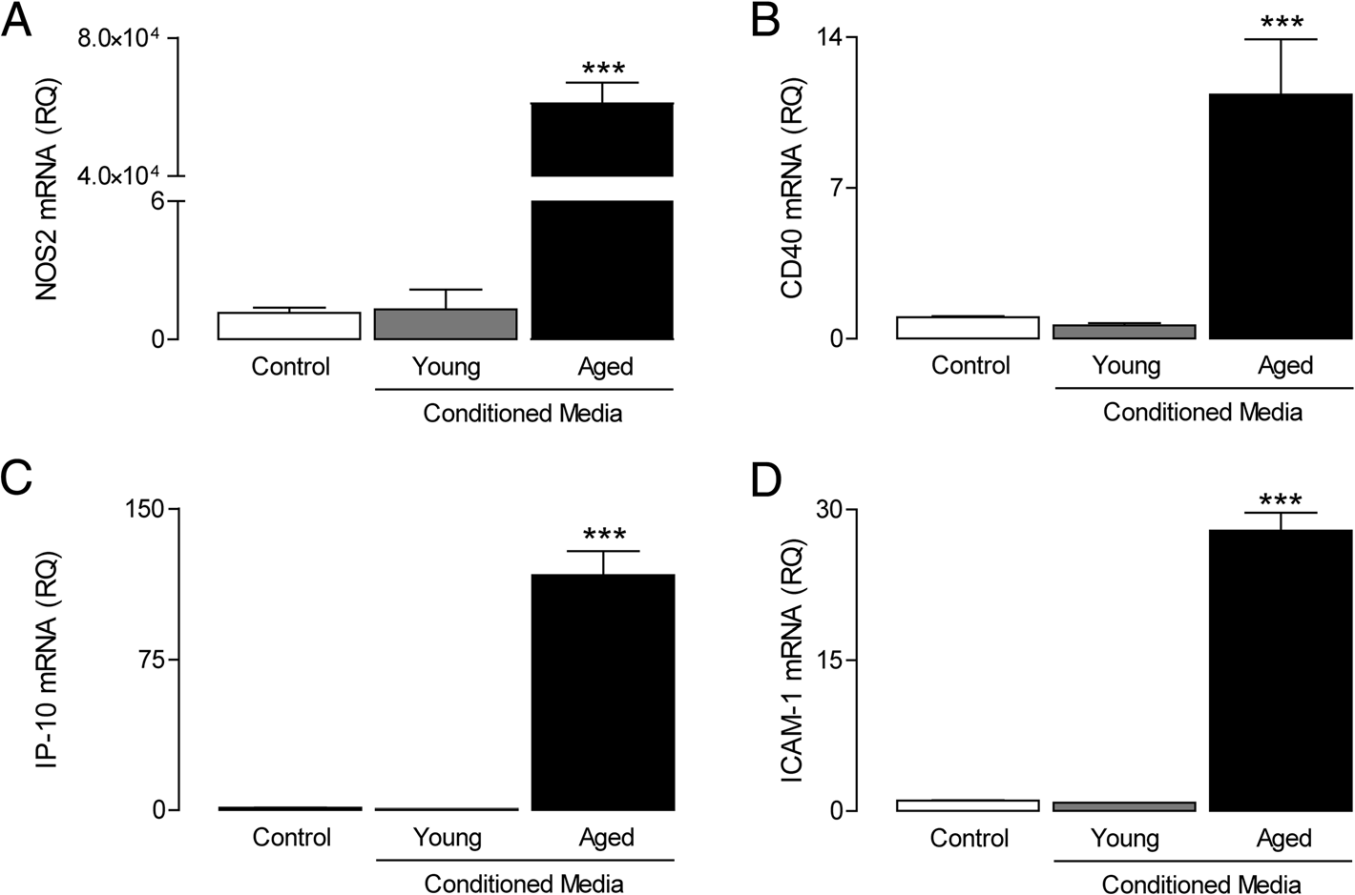
**Conditioned media derived from aged BMDMs induces glial activation.** Neonatal mixed glial cultures were incubated in the presence of conditioned media from unstimulated BMDMs derived from young and aged rats (24 h). Conditioned media from BMDMs derived from aged animals induced the expression of NOS2 **(A)**, CD40 **(B)**, IP-10 **(C)** and ICAM-1 mRNA (**(D)**; ^***^ *P* < 0.001; ANOVA), whereas exposure to conditioned media from BMDMs of young rats had no effect. Values are presented as means (±SEM; *n* = 3) expressed as a ratio to β-actin mRNA. NOS2, nitric oxide synthase; CD40, cluster of differentiation 40; IP-10, interferon gamma-induced protein 10; ICAM-1, intracellular adhesion molecule 1; RQ, relative quantities.

To evaluate whether BMDMs impact on glial activation, mixed glial cultures were incubated for 24 h in the presence of conditioned media obtained from unstimulated BMDMs of young and aged rats. Conditioned media prepared from BMDMs of aged rats induced a significant increase in mRNA expression of NOS2, CD40, IP-10 and ICAM1 (^*^P < 0.05; ^***^P < 0.001; ANOVA; Figure 5A, B, C, D), whereas conditioned media from BMDMs of young rats had no effect.

## Discussion

Inflammatory changes develop in the brain with age, and therefore, macrophages, which infiltrate the brain in increasing numbers with age, will respond to this microenvironment. We set out to model these conditions *in vitro* and compared the responses of BMDMs from aged and young rats to two potent inflammatory stimuli, LPS and IFNγ. We demonstrate that cells prepared from aged animals are more responsive to inflammatory stimuli, perhaps as a consequence of increased expression of TLR4 and IFNγR1, and show that conditioned medium obtained from BMDMs of aged rats increased microglial activation. This raises the possibility that infiltrating macrophages may contribute to the developing neuroinflammation.

Macrophages exhibit remarkable plasticity illustrated by their ability to adopt different phenotypes in response to different stimuli [2,33]. Predictably, in this study, LPS and IFNγ upregulated the expression of several markers of the classically activated phenotype including TNFα and NOS2, as well as cell surface markers of activation like CD40 and CD11b. The significant finding is that these stimuli induced a greater response in cells prepared from aged, compared with young, animals. The enhanced age-related LPS-induced response in respect of NOS2 mRNA contrasts with a previous finding in which the LPS-induced increase in cells from aged, compared with young, mice was not statistically significant [11]. The sensitivity of macrophages from aged, compared with young, rats to LPS extended to CD11b and CD40 mRNA, both of which showed age-related increases. Similarly, LPS increased TNFα release to a greater extent in BMDMs prepared from aged rats compared with young rats. The age-related increase in LPS-induced TNFα release was observed following 6 h of stimulation, but no age-related changes were observed following 3 h of treatment. This may be due to dysregulation of LPS-induced signalling. Significantly, TLR4 expression in BMDMs was increased with age, and this is one factor that may explain the increased age-related responses to LPS.

Like LPS, IFNγ triggered a greater increase in TNFα mRNA in BMDMs from aged, compared with young, rats, although this was not observed with NOS2 mRNA which is in broad agreement with an earlier report indicating that IFNγ-induced NO production was similar in BMDMs from aged and young mice [15]. The IFNγ-induced increase in mRNA expression of MHCII and CD40 was also greater in BMDMs from aged compared with young rats. This contrasts with the findings of Herrero and colleagues who reported that the IFNγ-induced effect on MHCII expression was decreased in aged mice [34]. While IFNγ-induced TNFα release from BMDMs is minimal, a significantly greater release was evident in BMDMs from aged rats at both timepoints assessed. The age-related increase in IFNγR expression in BMDMs provides one possible explanation for the increase in responsiveness to IFNγ.

Whereas relatively few studies have examined age-related changes in BMDMs, macrophages prepared from other tissues have been examined, and in the case of splenocytes, the evidence mainly suggests that there is an age-related decrease in responses to LPS [9,35]. Specifically, several groups have reported that the LPS-induced release of IL-1β, IL-6 and TNFα was decreased in splenocytes prepared from aged, compared with young, animals [11,12,36,37], although this is not universally accepted [10]. The latter group identified tissue-specific changes with an age-related increase in responsiveness of macrophages from spleen and alveoli to LPS and a decrease in peritoneal macrophages. Factors that may explain the refractory response include the reported age-related decrease in TLR4 expression on splenocytes [38] or the altered p38 MAPK signaling [36], or in the case of peritoneal macrophages [39], the increase in expression of the negative feedback regulator of TLR signaling, miR-146a [40].

The impetus driving this study was that an increase in infiltration of macrophages was observed with age [30] and in APP/PS1 mice [18], which may arise from the increased blood brain barrier permeability. Age-related changes in the permeability of the blood brain barrier (BBB) have been reported in rats [30], mice [41] and humans [42]. We have previously reported that BBB permeability is increased with age [30,31]. The present data confirm the age-related increase in infiltration of macrophages and increased expression of inflammatory molecules that include HMGB-1, IFNγ and TNFα which could potentially trigger activation of infiltrating macrophages. While some studies have suggested that resident brain cells are capable of producing IFNγ, it is generally believed that infiltrating peripheral cells are the source of IFNγ in the brain. Previous studies have demonstrated an age-related increase in the presence of CD3^+^ T cells [43], macrophages [30] and NK cells [44]. A recent study carried out by Baruch and colleagues suggests that decreased responsiveness to IFNγ signaling is a cause of cognitive dysfunction associated with aging [45], although we have shown that increased infiltration of macrophages in APP/PS1 mice is associated with increased hippocampal expression of IFNγ and a deficit in LTP [19]. We also report that there is an age-related increase in the chemokines MCP-1 and IP-10 which are chemoattractants for several cells including monocytes [32], and this, coupled with the increased BBB permeability, may contribute to the infiltration of cells. While a number of cell types present within the brain are capable of producing chemokines, it must also be considered that infiltrating monocytes may contribute to the increased expression of chemokines in the aged brain. Indeed, macrophages have been shown to upregulate the expression of these molecules in response to inflammatory stimuli [46].

It still remains unclear whether infiltrating macrophages play a beneficial or detrimental role within the CNS, with conflicting reports in the literature. On the one hand, they may be more efficient phagocytes than resident microglia [47] and therefore offer a level of protection. Consistent with this, and in the context of Alzheimer’s disease (AD), a deficiency of CCR2 in a mouse model of AD reduced monocyte trafficking into the CNS, and this was associated with increased Aβ plaque burden and mortality [48]. On the other hand, we have observed that infiltration of macrophages into the brain was associated with neuroinflammatory changes and loss of synaptic plasticity [20,30] and also progression of pathology in a mouse model of AD [18,19]. Similarly, monocyte infiltration correlated with progression to the paralytic stage in experimental allergic encephalitis, the mouse model of multiple sclerosis, whereas inhibiting monocyte recruitment to the CNS blocked disease progression [49]. The findings of a recent study also suggested that macrophages, irrespective of phenotype, were cytotoxic to cells in organotypic hippocampal slices [50]. Significantly, conditioned medium from BMDMs of aged rats increased expression of NOS2, CD40, IP-10 and ICAM1 in mixed glia leading us to propose that infiltrating macrophages have the capacity to contribute to the existing inflammatory environment in the brain with age.

The proposal is that, with age, changes in the blood brain barrier, coupled with upregulation of chemokines which are chemoattractant to macrophages, induce macrophage infiltration. These cells, which are more responsive to inflammatory stimuli, encounter a microenvironment which is characterized by increased expression of inflammatory mediators thereby triggering the cells to adopt the M1 phenotype and contributing to the age-related inflammatory cascade that is detrimental to brain function.
